# Supplementary material for: Sigma Factor Engineering in Actinoplanes sp. SE50/110: Expression of the Alternative Sigma Factor Gene ACSP50_0507 (σHAs) Enhances Acarbose Yield and Alters Cell Morphology
Source: Microorganisms. 2024 Jun 20;12(6):1241. doi: 10.3390/microorganisms12061241 (PMC11205660; doi:10.3390/microorganisms12061241)
Supplement: Supplementary file 1 [file microorganisms-12-01241-s001.zip › microorganisms-3024221-supplementary.pdf]

microorganisms

Article

Sigma Factor Engineering in *Actinoplanes* sp. SE50/110: Expression of the Alternative Sigma Factor Gene *ACSP50_0507* (σH^{As}) Enhances Acarbose Yield and Alters Cell Morphology

Laura Schlüter[1], Tobias Busche[2,3], Laila Bondzio[4], Andreas Hütten[4], Karsten Niehaus[5], Susanne Schneiker-Bekel[1,6], Alfred Pühler[6] and Jörn Kalinowski[1,2*]

1 Microbial Genomics and Biotechnology, Center for Biotechnology, Bielefeld University, 33594 Bielefeld Germany; lschluet@cebitec.uni-bielefeld.de (L.S.); schneike@cebitec.uni-bielefeld.de (S.S.-B.)
2 Technology Platform Genomics, Center for Biotechnology, Bielefeld University, 33594 Bielefeld, Germany; tbusche@cebitec.uni-bielefeld.de
3 Medical School East Westphalia-Lippe, Bielefeld University, 33594 Bielefeld, Germany
4 Faculty of Physics, Bielefeld University, 33594 Bielefeld Germany; laila.bondzio@uni-bielefeld.de (L.B.); andreas.huetten@uni-bielefeld.de (A.H.)
5 Proteome and Metabolome Research, Faculty of Biology, Bielefeld University, 33594 Bielefeld, Germany; kniehaus@cebitec.uni-bielefeld.de
6 Genome Research of Industrial Microorganisms, Center for Biotechnology (CeBiTec), Bielefeld University, 33594 Bielefeld, Germany; puehler@cebitec.uni-bielefeld.de

* Correspondence: joern@cebitec.uni-bielefeld.de

Table S1. Oligonucleotides used in this study.

Name	Sequence 5′ to 3′	Application
0507_pSETT4_fw	GGCACTAGTCGAGCAACGGAGGTATTCCGATGAC-CGTCACACAGACTTCGACG	Expression plasmid
0507_pSETT4_rev	GGCGGAAAATCACGCGGCACGAATCAGAGCGTCTCGGCGGC	Expression plasmid
pSETT4_fw	TGACCCCATGCCGAACTCAGAAGTGAAACG	Screening overexpression plasmid
pSETT4_rev	GTACTTCGTCGTGAAGGTCATGACACCATTATAACGAACG	Screening overexpression plasmid
gRNA_0507_fw	ACGCAACGGCCGAGGGAGTGGGTC	CRISPR/Cas gRNA
gRNA_0507_rev	AAACGACCCACTCCCTCGGCCGTT	CRISPR/Cas gRNA
0507_flank1_fw	TCGGTTGCCGCCGGGCGTTTTTTATCGAGGCGCGACGAGACTTCG	Deletion plasmid
0507_flank1_rev	CGACGACCGTCGACCGGGCCGCCGAGACGCTCTGAG	Deletion plasmid
0507_flank2_fw	CCTCAGAGCGTCTCGGCGGCCCGGTCGACGGTCGTCGAAG	Deletion plasmid
0507_flank2_rev	GCGGCCTTTTTACGGTTCCTGGCCTCCCACTGACCGCCGAAACG	Deletion plasmid
CRISPR_fw	TCGCCACCTCTGACTTGAGC	Screening deletion plasmid
CRISPR_rev	GGCGTTCCTGCAATTCTTAG	Screening deletion plasmid
gDNA_fw	GGCACTAGTCGAGCAACGGAGGTATTCCGATGGAC-GCAGGTAATGCCCG	RT-qPCR control for DNA
gDNA_rev	GGCGGAAAATCACGCGGCACGAATCAGGCGGCCTGCAGGC	RT-qPCR control for DNA
qPCR_0507_fw	GCTGATGCCGAGGTGGGTG	RT-qPCR 0507
qPCR_0507_rev	CATCATCGGCGAGATCAAGCG	RT-qPCR 0507
qPCR_5UTR_0507_fw	CCATCGCGTTGAGCAGTTCC	RT-qPCR 0507 5′UTR
qPCR _5UTR-0507_rev	GGTCCACCACACAGTGGACC	RT-qPCR 0507 5′UTR
pJOE_C-0507-His6_fw	TAATTTTCTTAAGAAGGAGATATACATATGAC-CGTCACACAGACTTCGACGACC	Protein expression with C-terminal His6 tag
pJOE_C-0507-His6_rev	CCAAGCTTAATGATGATGATGATGATGG-GATCCGAGCGTCTCGGCGGCCAGC	Protein expression with C-terminal His6 tag
EMSA_(Btn)_M-0507_fw	(X)ACGAGTGTCACTCATACTTTCGGCTAGTCTTTCGTCGGACAC-TCGGCTACGGTGGTCGCA	EMSA, motif of ACSP50-0507 (with and without 5′ biotinylation)
EMSA_M-0507_rev	TGCGACCACCGTAGCCGAGTGTCCGACGAAA-GACTAGCCGAAAGTATGAGTGACACTCGT	EMSA, motif of ACSP50-0507

EMSA-(Btn)_M-4135_fw	(X)GAAATTAGACTTTTGTTTTCTCTGTGATTACTGATCTTCGTG-GAGGGGATTAGAGCCCCCG	EMSA, motif of ACSP50-4135 (with and without 5′ biotinylation)
EMSA_M-4135_rev	CGGGGGCTCTAATCCCCTCCACGAAGATCAGTAATCACAGA-GAAAACAAAAGTCTAATTTC	EMSA, motif of ACSP50-4135
EMSA_(Btn)_neg_fw	(X)GGCTGCACCCGCGGCTGCCTGATCTGCCTGTCCCACATCAAGTG-CACGCCGAAGATGAAGA	EMSA, gLuc coding sequence as negative control (with and without 5′ biotinylation)
EMSA_neg_rev	TCTTCATCTTCGGCGTGCACTTGATGTGG-GACAGGCAGATCAGGCAGCCGCGGGTGCAGCC	EMSA, gLuc coding sequence as negative control

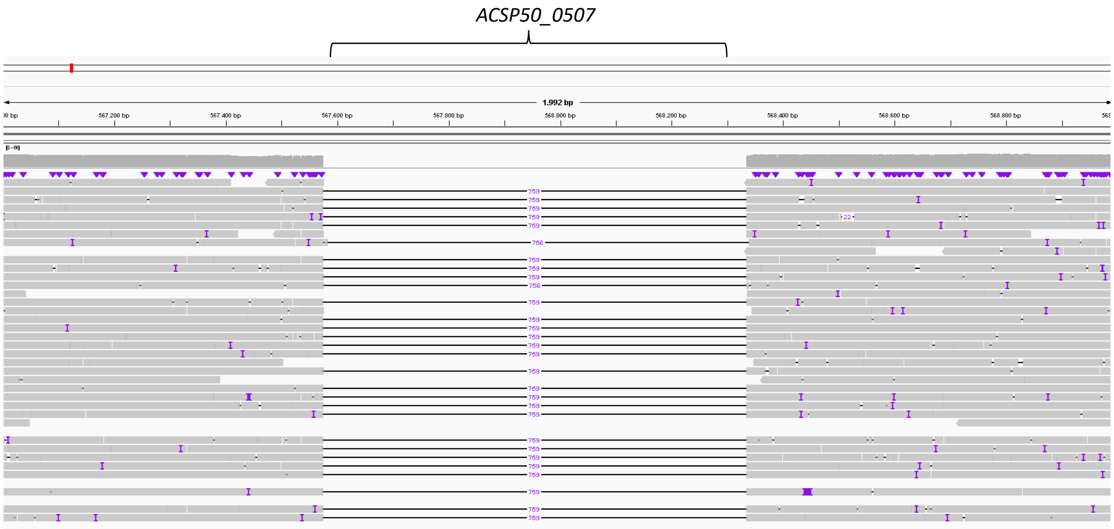

Figure S1. *sigH*^{As} deletion (Δ*sigH*^{As}) is verified by Nanopore whole genome sequencing. Integrative Genomics Viewer (IGV) (Robinson et al. 2011) view of a sequence deletion of 759 base pairs within the gene region of *sigH*^{As} is shown. Purple lines indicate insertions, which are caused by increased error rates during Nanopore sequencing due to homobase polymers. Reads were mapped using minimap2 (Li 2021), sorting and indexing was performed using samtools (Danecek et al. 2021).

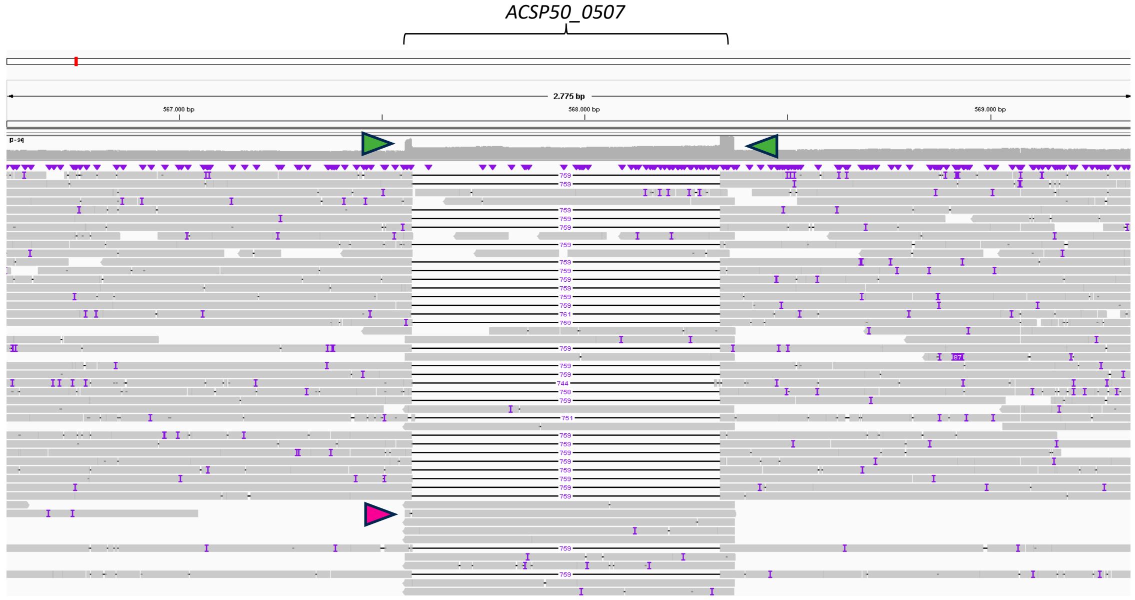

Figure S2. *sigH*^As^ complementation of Δ*sigH*^As^, based on the integrative pSET4*tipA*-*sigH*^As^ vector, is verified by Nanopore whole genome sequencing. IGV (Robinson et al. 2011) view of a gene deletion of a chromosomal gene copy and gene insertion at another position is shown. The sequence deletion of 759 base pairs within the gene region of *sigH*^As^ was confirmed. Further reads align to *sigH*^As^ without overlap to genomic surrounding, confirming the successful genomic integration of a second *sigH*^As^ copy in a different genome region (pink arrow). Due to gene deletion with approximately 30-50 base pairs on either side of the gene, accumulation of aligning sequences of these short regions due to the second gene copy are found (green arrow). Purple lines indicate insertions, which are caused by increased error rates during Nanopore sequencing due to homobase polymers. Reads were mapped using minimap2 (Li 2021), sorting and indexing was performed using samtools (Danecek et al. 2021).

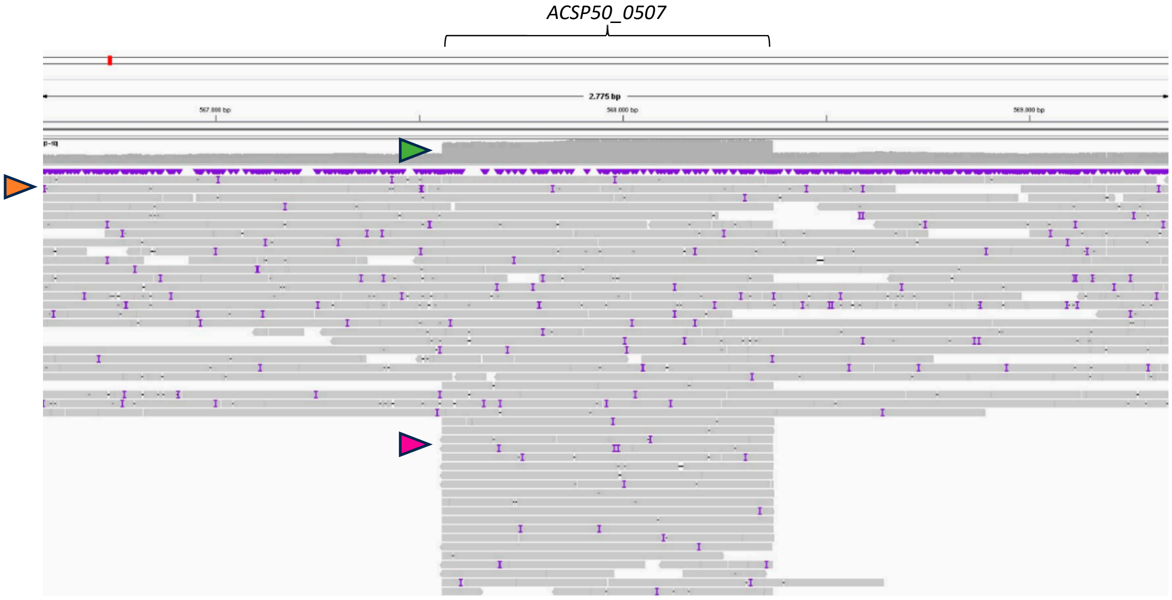

Figure S3. *sigH*^As^ expression, based on the integrative pSET4*tipA*-*sigH*^As^ vector, is verified by Nanopore whole genome sequencing. IGV (Robinson et al. 2011) view shows an increased number of reads at the gene region of *sigH*^As^, due to the integration of a second gene copy (green arrow). Presence of the chromosomal gene copy is confirmed by reads overlapping the gene environment (orange arrow) and the additional gene copy is confirmed by reads aligning to *sigH*^As^ but without any overlap to the genetic surrounding (pink arrow). Purple lines indicate insertions, which are caused by increased error rates during Nanopore sequencing due to homobase polymers. Reads were mapped using minimap2 (Li 2021), sorting and indexing was performed using samtools (Danecek et al. 2021).

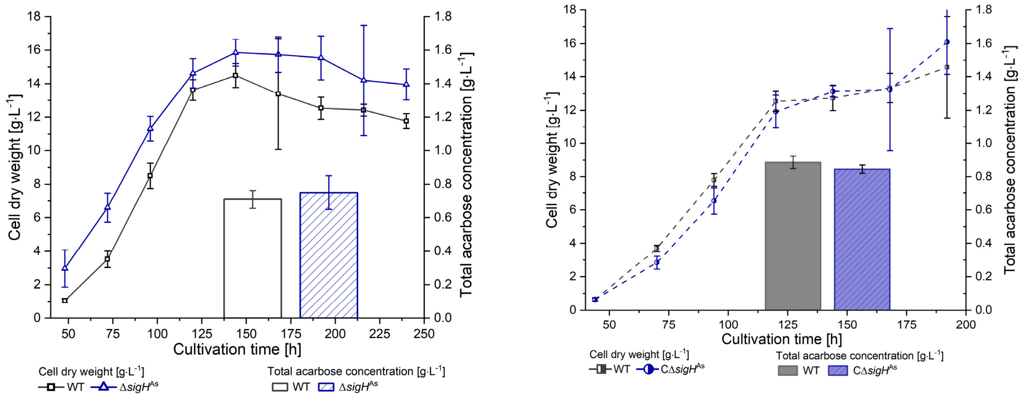

(**a**) (**b**)

Figure S4. Characterization of growth and acarbose production of *Actinoplanes* sp. SE50/110 sigma factor deletion and complementation mutants. (**a**) Cell dry weight (dense lines) and total acarbose concentration at 240 h of cultivation from supernatant in maltose minimal medium for *Actinoplanes* sp. SE50/110 wild type and deletion strain Δ*sigH*^{As} (n=3). (**b**) Cell dry weight (bracked lines) and total acarbose concentration at 192 h of cultivation from supernatant in maltose minimal medium for *Actinoplanes* sp. SE50/110 wild type and complementation strain CΔ*sigH*^{As} (n=3).

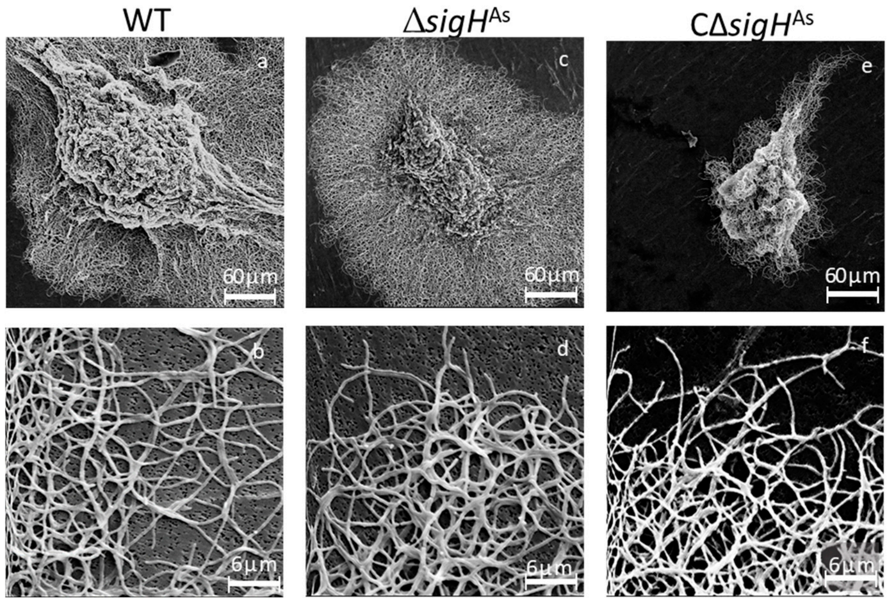

Figure S5. Scanning electron microscopy (SEM) of *Actinoplanes* sp. SE50/110 *sigH*^{As} deletion and complementation strain. Strains were cultivated in maltose minimal medium for 5 days before sample preparation for microscopic imaging. The wild type (**a**) grows as large, dense, mycelium, surrounded by a large network of hyphae. Hyphae of the wild type (**b**) have a uniform shape and are occasionally branched. *sigH*^{As} gene deletion had no influence on cell morphology (Δ*sigH*^{As}) (**c, d**) and gene complementation using the integrative vector pSETT4*tipA*-0507 had no visible effect (CΔ*sigH*^{As}) (**e, f**).

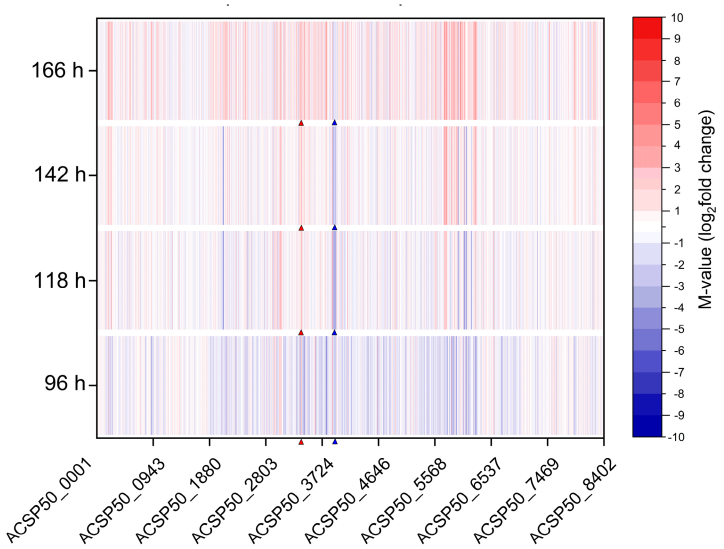

Figure S6. Transcriptomic analysis of *Actinoplanes* sp. SE50/110 *sigH*^{As} expression strain whole transcriptome. Comparative RNA-sequencing analysis of expression strain *sigH*^{As} to empty vector control strain pSETT4*tipA* for growth phase to late stationary phase in log2 fold change, indicated by colors as a heatmap. Arrowheads highlight putative metabolite gene cluster which are up- (red) or downregulated (blue).

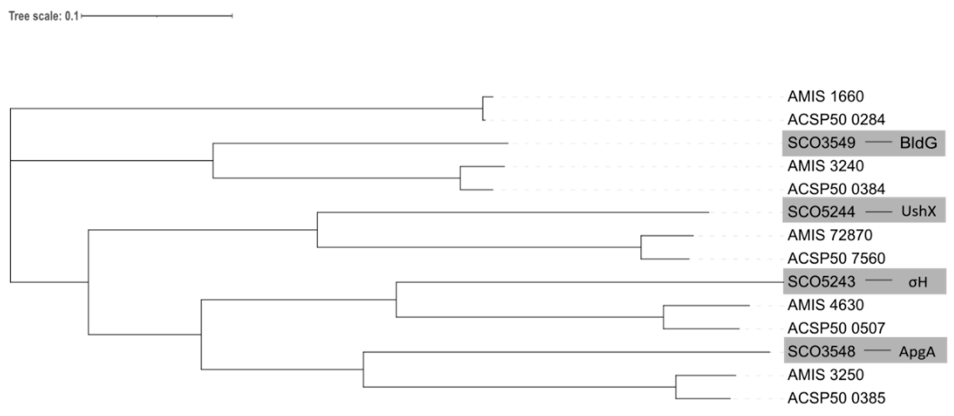

Figure S7. Phylogenetic tree of *Streptomyces coelicolor* σH^{Sc} regulatory proteins and their *Actinoplanes* sp. SE50/110 (ACSP50_) and *Actinoplanes missouriensis* (AMIS_) homologs. σH^{Sc} is inactivated by its anti-σ factor Ush, which can be inactivated by the anti-anti-σ factor BldG. Phylogenetic analysis was performed and visualized with ClustalW and iToL.

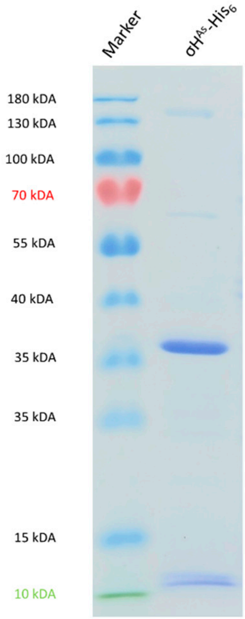

Figure S8. Purity of recombinant expressed σH^{As}-His$_6$ of ~29kDa analyzed using SDS-PAGE. Minor impurities were identified as the *E. coli* DNA-directed RNA-Polymerase subunits beta' at ~155 kDa and omega at ~12kDa as well as the ATP-dependent RNA helicase DeadD at ~65 kDa.

References

Danecek, Petr; Bonfield, James K.; Liddle, Jennifer; Marshall, John; Ohan, Valeriu; Pollard, Martin O. et al. (2021): Twelve years of SAMtools and BCFtools. In: *GigaScience* 10 (2). DOI: 10.1093/gigascience/giab008.

Li, Heng (2021): New strategies to improve minimap2 alignment accuracy. In: *Bioinformatics (Oxford, England)* 37 (23), S. 4572–4574. DOI: 10.1093/bioinformatics/btab705.

Robinson, James T.; Thorvaldsdóttir, Helga; Winckler, Wendy; Guttman, Mitchell; Lander, Eric S.; Getz, Gad; Mesirov, Jill P. (2011): Integrative genomics viewer. In: *Nature biotechnology* 29 (1), S. 24–26. DOI: 10.1038/nbt.1754.
